# Pleural fluid adenosine deaminase to serum C-reactive protein ratio for diagnosing tuberculous pleural effusion

**DOI:** 10.1186/s12890-023-02644-9

**Published:** 2023-09-15

**Authors:** Mohammad Fazle Rabbi, Mushfiq Newaz Ahmed, Md. Shafiqul Alam Patowary, Syed Rezaul Huq, S. M. Abdur Razzaque, Hossain Md. Arafat, Tasnuva Nahar, Mohammad Azmain Iktidar

**Affiliations:** 1 National Institute of Diseases of the Chest and Hospital, Dhaka, 1212 Bangladesh; 2 Dhaka Medical College Hospital, Dhaka, 1000 Bangladesh; 3 National Institute of Cancer Research and Hospital, Dhaka, 1212 Bangladesh; 4 Mugda Medical College Hospital, Dhaka, 1214 Bangladesh; 5 Directorate General of Health Services, Dhaka, Bangladesh; 6 School of Research, Chattogram, Bangladesh

**Keywords:** Pleural fluid ADA, Serum CRP, ADA to CRP ratio, Pleural effusion, Pleural tuberculosis

## Abstract

**Background:**

Tuberculous pleural effusion (TPE) and malignant pleural effusion (MPE) may occasionally show similar cytological and biochemical picture including ADA. In such cases, differentiating TPE and MPE is challenging and needs histopathology of pleural tissue which may involve invasive procedures. The present study aims to evaluate the diagnostic accuracy of pleural fluid ADA to serum CRP (ADA/CRP) ratio to discriminate between tuberculous and malignant pleural effusion. In addition, we investigated whether the ratio ADA/CRP adds diagnostic value to ADA.

**Methods:**

This cross-sectional study was conducted in the National Institute of Diseases of the Chest and Hospital (NIDCH), Mohakhali, Dhaka, from July 2021 to February 2022 on diagnosed patients of TPE and malignant pleural effusion MPE. A receiver operating characteristic curve (ROC) was constructed for identifying TPE. The added value of the ADA/CRP ratio to ADA was evaluated using the net reclassification improvement (NRI) and integrated discrimination improvement (IDI). A value of *p* < 0.05 was considered statistically significant for all tests.

**Results:**

Fifty-nine patients were enrolled in this study, of which 31 had TPE, and 28 had MPE. Pleural fluid ADA to serum CRP ratio and pleural fluid ADA level was significantly higher in patients with TPE, but there was no significant difference in serum CRP levels between patients with TPE and MPE. At cut off value of > 1.25, pleural fluid ADA to serum CRP ratio had a sensitivity of 93.8%, specificity of 85.2%, and positive and negative predictive values were 88.2% and 92% respectively, in the diagnosis of TPE and area under ROC curve (AUC) was 0.94. The NRI and IDI analyses revealed added diagnostic value of ADA/CRP to ADA.

**Conclusion:**

This study shows that the ADA/CRP ratio improves the diagnostic usefulness of ADA for TPE.

## Background

Pleural effusion, an abnormal accumulation of fluid within the pleural space, is not a disease itself but rather an important clinical manifestation of systemic and pleural diseases [1]. Internationally the incidence of pleural effusion is 320 cases per 100,000 people in industrialized countries [2] and only in the USA it is at least 1.5 million cases annually [3]. Pleural effusion has several etiologies, which are usually grouped into two categories: exudative and transudative. Two of the most important causes of exudative effusions are tuberculosis (TB) and malignancy [4].

Tuberculosis occurs in every part of the world, but most cases and deaths are registered in developing countries. Nearly 10.6 million people around the world developed TB in 2021, with an estimated 1.6 million deaths [5]. One of the most common presentations for extrapulmonary TB is tuberculous pleural effusion. Direct smears of pleural effusion or effusion cultures are often negative [6]. Pleural fluid (PF) AFB smears are positive in only < 5% of cases, and culture is positive in only 10–20% of cases [7]. Although pleural biopsy for histopathology is 80–100% sensitive in diagnosing tuberculous pleural effusion and 42–97% sensitive in diagnosing malignant pleural effusion, the procedure itself is invasive and requires expert and trained human resources [7].

Due to their low cost, and short turn-around time, PF biomarkers serve as complementary diagnostic tools. Adenosine deaminase (ADA) is one such commonly explored investigation tool in the case of tuberculous pleural effusion (TPE)with 90% sensitivity and a 92% specificity [8]. But ADA level can also be raised in malignancy, lymphoma, and collagen vascular disease [9]. Ogata et al. (2011) also demonstrated that although ADA activity in pleural fluid is highly sensitive (85.5%) and specific (86.5%) in the diagnosis of TPE; lung cancer, or mesothelioma may show high ADA activity [10]. Given ADA's inadequate diagnostic accuracy, it is still important to develop novel biomarkers to improve or replace it [11]. C-reactive protein (CRP) was discovered in 1930 and is widely used as a sensitive but nonspecific marker of systemic inflammation [12]. Increased serum C-reactive protein levels have been reported in many pulmonary disorders, including pneumonia, malignancies, and pulmonary thromboembolism [13]. Although few studies reported differences in serum CRP levels between tuberculous and malignant effusion, serum CRP alone is not a reliable marker to differentiate between these two types of effusion [13].

Pleural fluid ADA to serum CRP ratio (ADA/CRP) can be a novel, cost-effective tool in differentiating malignant from tuberculous pleural effusion; however, there is insufficient and contradictory evidence regarding its effectiveness [14, 15]. So, the present study aims to evaluate the diagnostic accuracy of pleural fluid ADA to serum CRP ratio to discriminate between tuberculous and malignant pleural effusion. In addition, we investigated whether the ratio of ADA to CRP adds diagnostic value to ADA.

## Materials and methods

### Study design, site and duration

This cross-sectional study was conducted from July 2021 to February 2022. Department of Respiratory Medicine of the National Institute of Diseases of the Chest and Hospital (NIDCH), Mohakhali, Dhaka, Bangladesh, was selected as the study site as it is the highest referral center for chest diseases and receives patients from all over the country.

### Study participants

Patients with tuberculous pleural effusion diagnosed based on histopathology of pleural biopsy or patients with malignant pleural effusion diagnosed based on pleural fluid cytology for malignant cells or histopathology of pleural biopsy and providing consent were included in the study. Patients with transudative pleural effusion, congestive heart failure, chronic liver disease, chronic kidney disease, and connective tissue diseases were excluded from the study.

### Sample size calculation and sampling method

Sample size was calculated using Buderer’s formula [16] where ${Z}_{1-\alpha /2}$ = 1.96 (for 95% confidence interval), sensitivity of ADA/CRP = 79% [14], specificity of ADA/CRP = 83% [14], prevalence of TPE = 68.7% [11], and absolute precision = 18%. Sample size based on the anticipated specificity of ADA/CRP (54 patients) was greater than that of the anticipated sensitivity (29 patients), therefore the former was chosen.

**Fig. 1:**
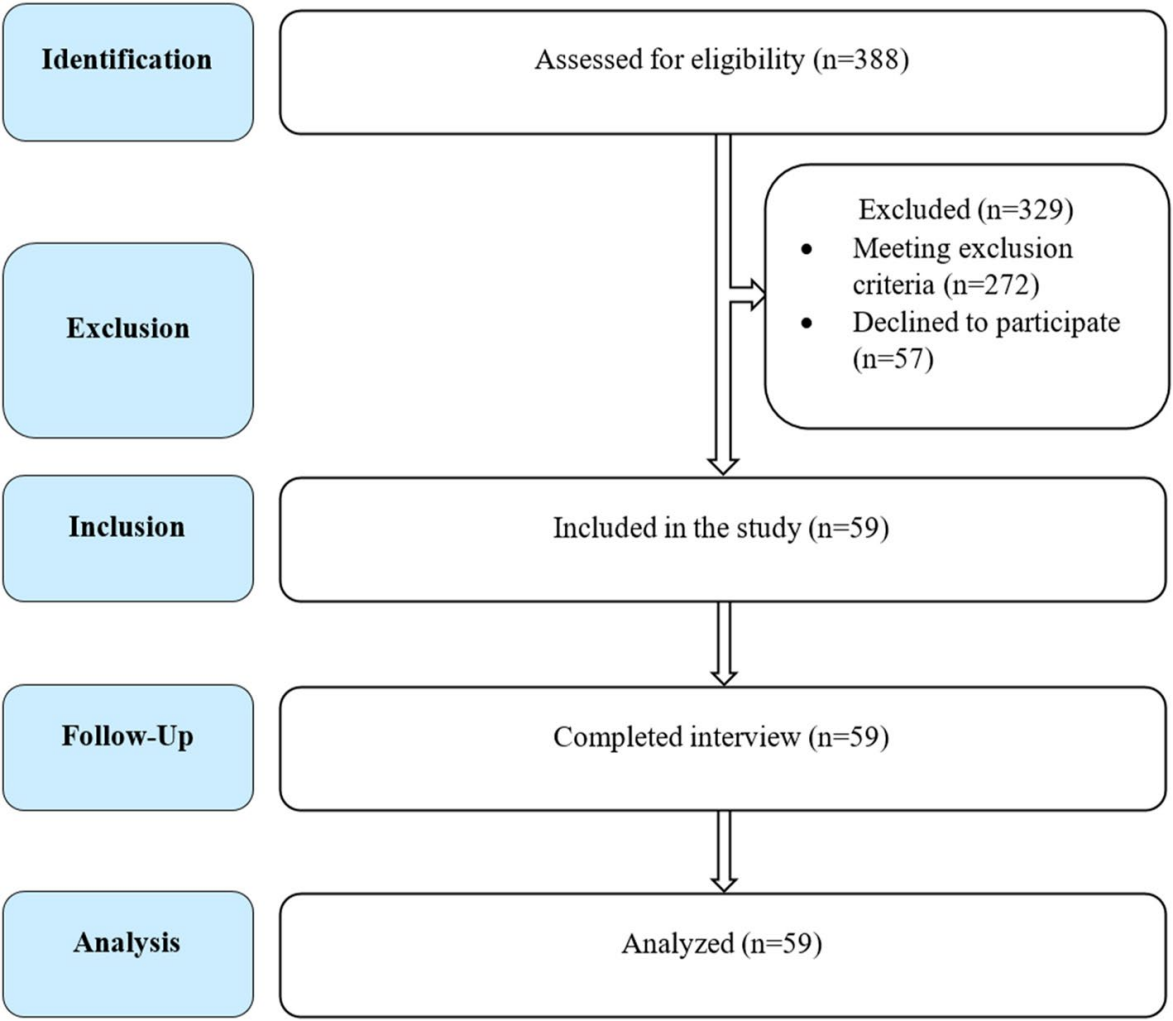
A flow chart of study participants at different stages of the study

All the newly admitted patients with unilateral pleural effusion (evident from history, clinical examination and chest X-ray poster-anterior view) during the study period were consecutively approached for enrolment in the study. Among whom, 272 were excluded for meeting one or more of the exclusion criteria, and 57 were excluded as they/their guardian didn't provide consent to enter the study, leaving a final sample size of 59 (Fig. 1).

### Operational definitions

Abnormal fluid accumulation between the parietal and visceral pleura is defined as pleural effusion [1]. If pleural fluid protein exceeded 3g/dl, it was considered exudative pleural effusion [17]. Tuberculous Pleural Effusion was diagnosed by the detection of caseating granuloma in pleural biopsy specimen. Malignant Pleural Effusion was diagnosed by cytological observation of malignant cells in pleural fluid or histological confirmation of malignancy in pleural biopsy specimen. Pack years of smoking are calculated by multiplying the number of packs of cigarettes smoked per day by the number of years the person has smoked (where a cigarette pack is calculated as the number of cigarettes/20) [18]. Someone who has smoked more than 100 cigarettes in their lifetime and has smoked in the last 28 days was considered a current smoker [19]. An individual who has smoked more than 100 cigarettes in their lifetime and has not smoked in the last 28 days were considered ex-smoker [19]. A person who has smoked no more than 100 cigarettes in their lifetime and does not currently smoke was defined as never smoker [19].

### Data collection

Data were collected through face-to-face interviews of patients/guardians using a pretested structured questionnaire. Background information, previous medical records, physical findings, and laboratory reports were recorded. After receiving informed consent, diagnostic thoracocentesis was performed on all patients. Once the thoracocentesis site has been identified, the skin encompassing the site is thoroughly rinsed with an antiseptic solution. Then, 2% xylocaine is used to administer local anesthesia to the skin, subcutaneous tissue, muscles, and parietal pleura. Then, a 20-cc syringe with a 22 G needle is inserted into the intercostal space at the upper border of the lower rib, and 10 to 20 cc of pleural fluid is aspirated. Pleural fluid was then sent for pleural fluid study, including physical appearance, biochemistry (protein and glucose), cytology, exfoliative cytology for malignant cell, Gene Xpert for MTB and ADA. Pleural biopsy was performed using Abrams pleural biopsy needle with all aseptic precautions followed by histopathological examination. Blood was collected and analyzed for serum CRP. Echocardiography was performed to rule out heart failure, ascitic fluid analysis was performed to determine the cause of ascites when associated with pleural effusion, blood urea, and serum creatinine were measured to rule out renal failure as a cause of pleural effusion, and a thyroid function test was performed to rule out hypothyroidism as a cause of pleural effusion.

### Data processing and analysis

Using Microsoft Excel, collected data were cleansed, validated, and encoded. For data analysis, we used Stata (version 16; StataCorp, College Station, TX, USA). Using a histogram, a normal Q-Q plot, and the Kolmogorov–Smirnov test, the normality of continuous data was determined. As a measure of the center of quantitative data, the arithmetic means and the median was used, while the standard deviation and interquartile range were used as a measure of dispersion. We summarized qualitative data using frequency and relative frequency. When applicable, the chi-square test and t-test were used to examine associations between predictor and outcome variables. A receiver operating characteristic (ROC) curve was plotted to evaluate the sensitivity, specificity, and area under the curves (AUC) with a 95% confidence interval (CI) to measure the efficacy level of the ratio of pleural fluid ADA to serum CRP, pleural fluid ADA and serum CRP for the diagnosis of TPE. We determined the added value of the pleural fluid ADA to serum CRP ratio to ADA using the net reclassification improvement (NRI) and integrated discrimination improvement (IDI) [20]. A two-tailed *p*-value of less than 0.05 was deemed statistically significant. All the reporting was done according to the Standards for Reporting of Diagnostic Accuracy Studies (STARD) guideline [21].

## Results

A total of 59 patients participated in the study, among which the majority (52.5%) had tuberculous pleural effusion, and the rest (47.5%) of the respondents had malignant pleural effusion. Table 1 shows that the average age of the entire study cohort was 44.39 years, and the mean age of patients with tuberculous pleural effusion was significantly lower than that of patients with malignant pleural effusion (TPE: 35.7 years, MPE: 53.9 years). The participants reported an average of 22.77 pack years of smoking, and those with MPE had a significantly higher average pack-year smoking history (TPE: 18.5 pack year, MPE: 25.8 pack year). The preponderance of participants were Muslim (94%) and female (67.8%). Tuberculous pleural effusion patients had a significantly higher median PF ADA (TPE: 57 U/L, MPE: 17.1 U/L) and PF ADA to serum CRP ratio (TPE: 2.16, MPE: 0.48) than MPE patients.

**Table 1:** Patient characteristics and types of pleural effusion (*N* = 59)

Variables	Entire Study Cohort		Tuberculous Pleural Effusion (*n* = 31, 52.5%)		Malignant Pleural Effusion (*n* = 28, 47.5%)		*p*-value
**Sociodemographic Variables**							
Age (in years), mean (SD)	44.39 (20.91)		35.8	(21.4)	53.9	(15.8)	< 0.001
Cigarette smoked (pack year), mean (SD)	22.77 (10.1)		18.5	(11.2)	25.8	(8.3)	0.045
Gender							0.9
Female	40	(67.8%)	21	(52.5%)	19	(47.5%)	
Male	19	(32.2%)	10	(52.6%)	9	(47.4%)	
Religion							0.09
Islam	56	(94.9%)	28	(50.0%)	28	(50.0%)	
Other	3	(5.1%)	3	(100.0%)			
Smoking History							0.2
Non-smoker	28	(47.5%)	18	(64.3%)	10	(35.7%)	
Ex-Smoker	6	(10.2%)	2	(33.3%)	4	(66.7%)	
Smoker	25	(42.4%)	11	(44.0%)	14	(56.0%)	
Residence							0.003
Rural	30	(50.8%)	10	(33.3%)	20	(66.7%)	
Urban	29	(49.2%)	21	(72.4%)	8	(27.6%)	
**Clinical Features**							
Weight loss	52	(88.1%)	28	90.3	24	85.7	0.585
Cough	52	(88.1%)	24	77.4	28	100.0	0.007
Fever	34	(57.6%)	31	100.0	3	10.7	0.001
Chest pain	34	(57.6%)	10	32.3	24	85.7	0.001
Hemoptysis	16	(27.1%)	3	9.7	13	46.4	0.002
Lymphadenopathy	7	(11.9%)					
No			31	100.0	21	75.0	0.012
Yes			0	0.0	7	25	
**Laboratory Parameters**							
PF WBC total count (cell/cmm), median (IQR)	300.0 (85.0–1400.0)		750.0 (200.0–1850.0)		155.0 (57.5–387.0)		0.008
Cellular predominance							
Lymphocyte	52	(88.1%)	27	87.1	25	89.3	0.795
Neutrophil	7	(11.9%)	4	12.9	3	10.7	
PF ADA (U/L), median (IQR)	40.0 (17.2–60.0)		57.0 (43.6–75.0)		17.1 (12.1–22.6)		< 0.001
Serum CRP (mg/L), median (IQR)	30.0 (15.7–51.7)		30.0 (18.2–34.7)		34.0 (14.3–64.0)		0.316
PF ADA to serum CRP ratio, median (IQR)	1.38 (0.49–2.32)		2.16 (1.49–3.35)		0.48 (0.24–1.14)		< 0.001

Values are expressed as n (%) unless otherwise mentioned

*Abbreviations: ADA* adenosine deaminase, *CRP* C-reactive protein, *IQR* inter-quartile range, *PF* pleural fluid, *SD* standard deviation, *WBC* white blood cell

**Fig. 2:**
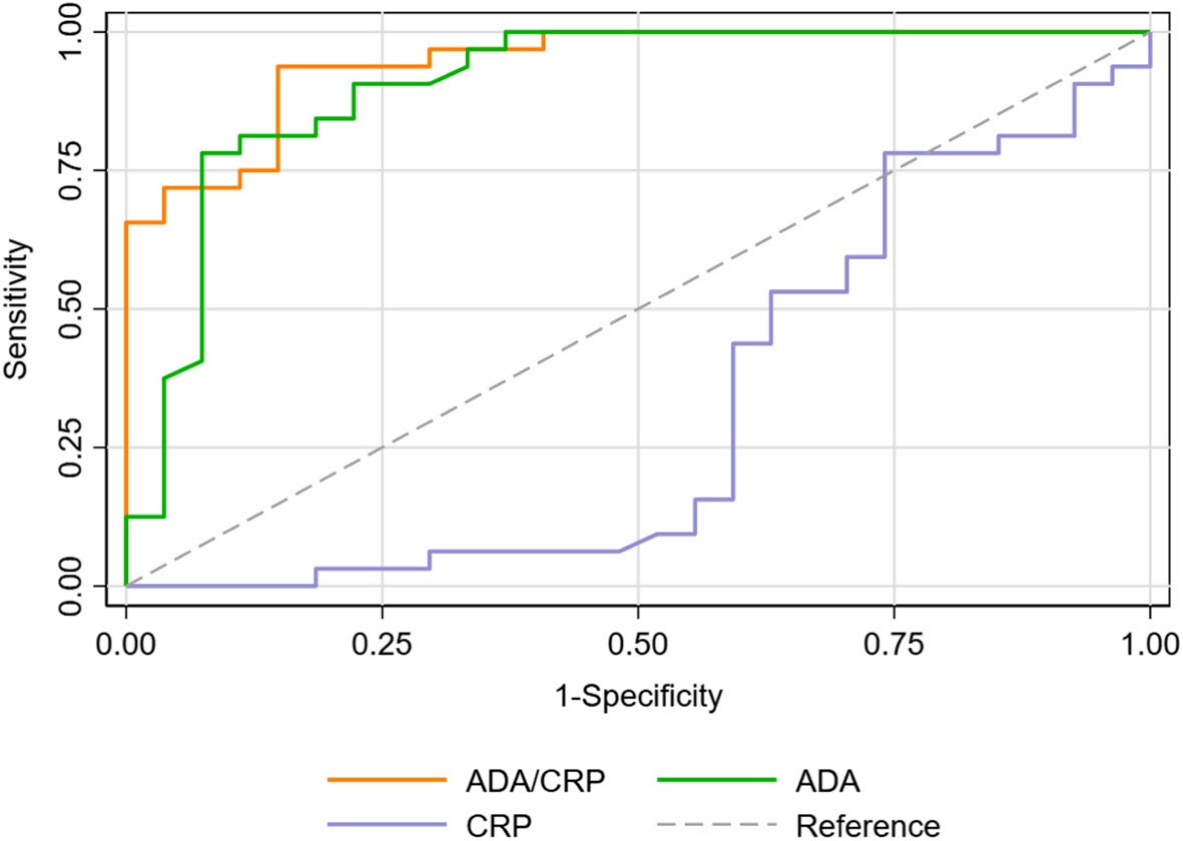
Receiver operating characteristic (ROC) curve of pleural fluid ADA to serum CRP ratio, pleural fluid ADA and serum CRP. Area under the ROC curves for ADA/CRP: 0.94 (95% confidence interval [CI], 0.89–0.99), for ADA: 0.90 (95% confidence interval [CI], 0.82–0.98), for CRP: 0.32 (95% confidence interval [CI], 0.17–0.46)

The receiver operating characteristic (ROC) curve for pleural fluid ADA to serum CRP ratio, pleural fluid ADA and serum CRP is depicted in Fig. 2. The area under the curve (AUC) for both the ADA/CRP (0.94) and ADA was high (0.90), and the *p*-value was highly significant (< 0.001). Both the lower and upper bound area was also above the area of 0.5, indicating that both ADA/CRP and ADA could accurately predict TPE; among them, ADA/CRP is better. The AUC of CRP was 0.32 (95% confidence interval [CI], 0.17–0.46), and the upper and lower bound of the 95% confidence interval was below 0.5. Therefore, serum CRP is a poor predictor of TPE.

Table 2 shows that according to the Youden index, at maximum J value (78.9%), the best cut-off value of pleural fluid ADA to serum CRP ratio was 1.25. At a cut-off value of ≥ 1.25, pleural fluid ADA to serum CRP ratio had a sensitivity of 93.8%, specificity of 85.2%, positive and negative predictive values of 88.2% and 92%, respectively. On the other hand, at maximum J value (70.1%), the best cut-off value of pleural fluid ADA to serum CRP ratio was 40.3 U/L. At a cut-off value of ≥ 40.3 U/L, pleural fluid ADA had a sensitivity of 81.3%, specificity of 88.9%, and positive and negative predictive values were 89.7% and 80%, respectively.

**Table 2:** Sensitivity, specificity, Youden index, PLR, NLR, PPV, and NPV of pleural fluid ADA to serum CRP ratio and pleural fluid ADA in diagnosing patients with tuberculous pleural effusion

	**Cut-off**	**Sensitivity (%)**	**Specificity (%)**	**Youden index (%)**	**PPV (%)**	**NPV (%)**	**PLR**	**NLR**
ADA/CRP	1.25	93.8	85.2	78.9	88.2	92	6.33	0.07
ADA (U/L)	40.3	81.3	88.9	70.1	89.7	80.0	7.31	0.21

*PLR* positive likelihood ratio, *PPV* positive predictive value, *NLR* negative likelihood ratio, *NPV* negative predictive value

Youden Index, J = max (sensitivity + specificity-1)

Table 3 contains the outcomes of net Reclassification Improvement (NRI) and integrated Discrimination Improvement (IDI) analyses. We observed statistically significant NRI and IDI (*p* < 0.05). These findings suggest that the ADA/CRP ratio adds diagnostic value to ADA.

**Table 3:** Net Reclassification Improvement (NRI) and Integrated Discrimination Improvement (IDI) Analysis

Continuous NRI		IDI	
**Estimates (95% CI)**	***p*-value**	**Estimates (95% CI)**	***p*-value**
1.12 (0.26, 4.28)	< 0.001	0.17 (0.06, 0.27)	0.002

*IDI* integrated discrimination improvement, *NRI* net reclassification improvement

## Discussion

This study was carried out to assess the validity of pleural fluid ADA to serum CRP ratio for differentiation of tuberculous from malignant pleural effusion. While prior research investigated the diagnostic accuracy of the pleural fluid ADA to serum CRP ratio for TPE [14, 15, 22], they did not assess the ratio's additional value over ADA alone. This is, to the best of our knowledge, the first study to look at the diagnostic significance of the ADA/CRP ratio in addition to ADA. This study shows that the ADA/CRP ratio improves the diagnostic usefulness of ADA for TPE.

In this study, a significantly higher level of pleural fluid ADA was found in TPE in comparison to MPE (median 57 U/L vs. 17.1 U/L, *p* < 0.001) which is consistent with Ernam et al. (2005) (median 75.41 U/L vs. 22.09 U/L, *p* < 0.001) [23]. A retrospective study over 2100 patients revealed that at a cut-off value of > 35 U/L, pleural fluid ADA had 93% sensitivity, and 90% specificity in diagnosing TPE [24] In our study, at a cut-off value of ≥ 40.3, pleural fluid ADA had a lower sensitivity of 81.3%, and similar specificity of 88.9%. However, conflicting data were obtained by Zarić et al. (2008), who reported poor specificity (70.4%), despite acceptable sensitivity (89.2%) of ADA at a cut-off value of 49 U/L in diagnosing TPE [25].

Patients with TPE have significantly higher levels of ADA/CRP ratio compared to patients with MPE (median 2.16 vs 0.48, *p* < 0.001). A concordant finding was also found in Swetha et al. [14], Kadhim and Hashim [22] and Venkatesh et al. [15], where significantly higher ADA/CRP ratio in the TPE group in comparison to the MPE [14]. In the present study, area under the ROC curve was 0.94 (95% confidence interval [CI], 0.89–0.99), which was statistically significant (*p* < 0.001) and a cut-off ≥ 1.25 showed 93.8% sensitivity and 85.2% specificity. These findings are corroborated by the findings of Swetha et al. [14]. They reported that at cut-off value of ≤ 1.2, pleural fluid ADA: serum CRP ratio was 78.95% sensitive and 83.33% specific in differentiating patients with MPE from TPE, yielding an AUC of 0.789 on ROC [14].

In our study, the AUC of the ADA/CRP ratio was better than that of the ADA (0.94 vs 0.90). We employed the NRI and IDI to determine whether the ADA/CRP ratio offers additional diagnostic value beyond ADA because the AUC of ROC has certain limitations in determining the overall diagnostic accuracy of a given test [26]. These two statistical techniques are frequently employed to determine the additional diagnostic value of a certain diagnostic model [20]. Both IDI and continuous NRI of the ADA/CRP ratio were greater than 0, and the corresponding *p*-values were < 0.05. Thus, we concluded that the ADA/CRP ratio increases the diagnostic precision of ADA.

Therefore, ADA/CRP ration can be an efficient and cost-effective tool to differentiate TPE from MPE in clinically perplexed situations as these two diseases often present with similar clinical pictures. Before opting for a more expensive and invasive procedure which is also often difficult to obtain in resource-limited healthcare settings, this tool can bolster the clinical impression of primary care physicians and in difficult-to-refer cases give them the confidence to initiate anti-TB medication. However, it is also important not to forget the role of gold standard tests for the diagnosis of TPE and MPE.

Even though this is one of the first studies to look at the additional diagnostic value of ADA/CRP over ADA, it has some limitations. Our sample size was relatively smaller and further comprehensive studies with larger sample sizes are required to validate our findings. Moreover, we only included tuberculous and malignant pleural effusion cases. The inclusion of other causes of exudative effusion in the study would have provided a more comprehensive finding. Tissue culture was not done in the study due to the long turn-around time, which would have delayed the treatment of the patient and complicate the case. As our country is one of the top TB burden countries, if we have a patient suggestive of signs and symptoms of TB who demonstrated caseating granuloma in pleural biopsy, we can make a diagnosis of TPE as per our national guideline [27].

## Conclusion

Although both pleural fluid ADA to serum CRP ratio and pleural fluid ADA are useful tools in differentiating between tuberculous and malignant pleural effusion, ADA/CRP ratio has added diagnostic value over ADA. A cut-off value of ≥ 1.25 is identified as the optimal cut-off value for ADA/CRP ratio, with 93.8% sensitivity and 85.2% specificity. ADA/CRP can therefore help to differentiate TPE from MPE in clinically puzzling scenarios, especially in resource-limited settings.

## Data Availability

The dataset used and/or analysed during the current study are available from the corresponding author on reasonable request.
