# Correction to: Pleural fluid adenosine deaminase to serum C-reactive protein ratio for diagnosing tuberculous pleural effusion

**DOI:** 10.1186/s12890-023-02694-z

**Published:** 2023-10-09

**Authors:** Mohammad Fazle Rabbi, Mushfiq Newaz Ahmed, Md. Shafiqul Alam Patowary, Syed Rezaul Huq, S. M. Abdur Razzaque, Hossain Md. Arafat, Tasnuva Nahar, Mohammad Azmain Iktidar

**Affiliations:** 1 National Institute of Diseases of the Chest and Hospital, Dhaka, 1212 Bangladesh; 2 Dhaka Medical College Hospital, Dhaka, 1000 Bangladesh; 3 National Institute of Cancer Research and Hospital, Dhaka, 1212 Bangladesh; 4 Mugda Medical College Hospital, Dhaka, 1214 Bangladesh; 5 Directorate General of Health Services, Dhaka, Bangladesh; 6 School of Research, Chattogram, Bangladesh

**Correction to: BMC Pulmonary Medicine (2023) 23:349**

10.1186/s12890-023-02644-9

Following publication of the original article, the authors flagged that the name of the fourth author had been incorrectly spelled as ‘Syed Rezaul Haque’ in place of ‘Syed Rezaul Huq’. The author list has since been corrected in the article. The authors thank you for reading this erratum and apologize for any inconvenience caused.

